# The first versatile human iPSC-based model of ectopic virus induction allows new insights in RNA-virus disease

**DOI:** 10.1038/s41598-020-72966-9

**Published:** 2020-10-08

**Authors:** Stefan Peischard, Huyen Tran Ho, Ilaria Piccini, Nathalie Strutz-Seebohm, Albrecht Röpke, Ivan Liashkovich, Hiteshika Gosain, Bettina Rieger, Karin Klingel, Britta Eggers, Katrin Marcus, Wolfgang A. Linke, Frank Ulrich Müller, Stephan Ludwig, Boris Greber, Karin Busch, Guiscard Seebohm

**Affiliations:** 1 Institute for Genetics of Heart Diseases (IfGH), Department of Cardiovascular Medicine, University Hospital Münster, 48149 Münster, Germany; 2 Interdisciplinary Centre for Clinical Research (IZKF), Faculty of Medicine, University of Münster, 48149 Münster, Germany; 3 Human Stem Cell Pluripotency Laboratory, Max Planck Institute for Molecular Biomedicine, 48149 Münster, Germany; 4 Chemical Genomics Centre of the Max Planck Society, 44227 Dortmund, Germany; 5 Institute of Human Genetics, University Hospital Münster, 48149 Münster, Germany; 6 Institute of Physiology II, Faculty of Medicine, University of Münster, 48149 Münster, Germany; 7 Institute of Molecular Cell Biology, Department of Biology, University of Münster, 48149 Münster, Germany; 8 Cardiopathology, Institute for Pathology and Neuropathology, University Hospital Tübingen, Liebermeisterstrasse 8, 72076 Tübingen, Germany; 9 Faculty of Medicine, Medizinisches Proteom-Center, Ruhr-University Bochum, 44801 Bochum, Germany; 10 Institute of Pharmacology and Toxicology, University of Münster, 48149 Münster, Germany; 11 Institute of Virology Muenster (IVM), Centre for Molecular Biology of Inflammation (ZMBE), University of Münster, 48149 Münster, Germany; 12 Present Address: RheinCell Therapeutics GmbH, 40764 Langenfeld, Germany

**Keywords:** Cardiovascular diseases, Stem-cell research, Viral infection

## Abstract

A detailed description of pathophysiological effects that viruses exert on their host is still challenging. For the first time, we report a highly controllable viral expression model based on an iPS-cell line from a healthy human donor. The established viral model system enables a dose-dependent and highly localized RNA-virus expression in a fully controllable environment, giving rise for new applications for the scientific community.

## Introduction

**Figure 1:**
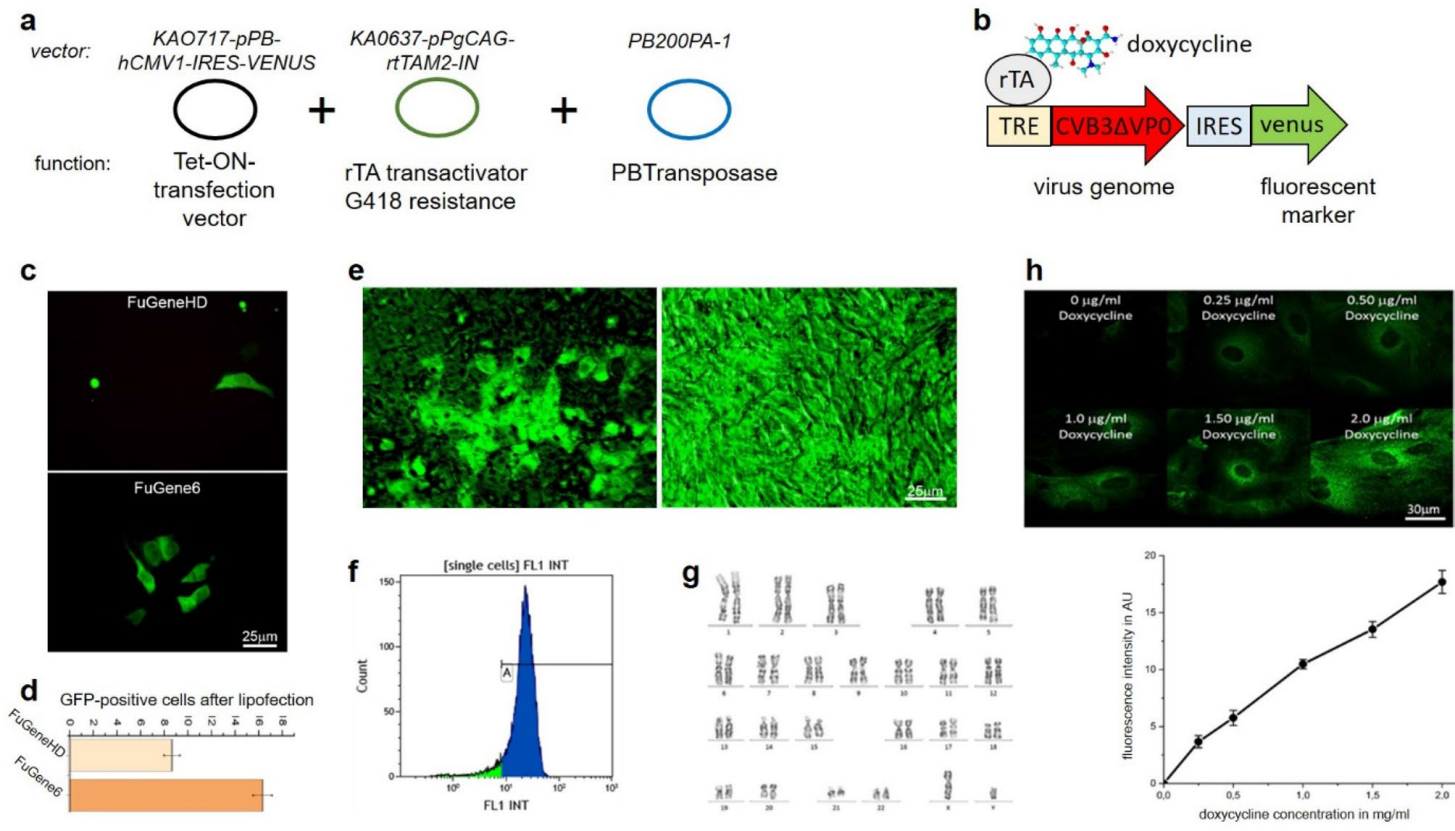
Development of a controllably inducible CVB3 human iPSC line for humans. (**a**) A triple-vector system was designed for the generation of a stable transfected iPSC-line expressing CVB3ΔVP0 dependent on the doxycycline concentration present in the cell culture medium. Triple-vector system: Co-transfection of vector KAO717-pPB-hCMV1-IRES-Venus with inserted CVB3ΔVP0, vector KA0637-pPgCAG-rtTAM2-IN and vector of PB200PA-1. (**b**) The expected transgenic line contains the CVB3∆VP0 virus genome and a fluorescent Venus marker. Their common expression is controlled by doxycycline application. (**c**) Comparative transfection of 150,000 SFS.1 cells with FuGeneHD or FuGene6 to identify the optimal transfection reagent for huge constructs in this iPS-cell line. Fluorescence images to determine the relative efficiency of the transfections using a cytosolic GFP construct. (**d**) Quantification of transfection efficiency by FACS analysis of pcDNA-CrispR-GFP transfected cells. Error bars represent ± SEM calculated from three independent FACS sortings (n = 3) each. (**e**) Successfully transfected SFS.1-CVB3ΔVP0-IRES-Venus colony expressing a clearly visible Venus marker three days after the first doxycycline application (left). Purified SFS.1-CVB3ΔVP0-IRES-Venus cell colony obtained after two further picking and reseeding steps to ensure the generation of a homogenously CVB3ΔVP0 expressing cell-line (right). (**f**) FACS analysis of purified SFS.1-CVB3ΔVP0-IRES-Venus cells shows almost 100% Venus expressing cells in the cell suspension. (**g**) Karyogram of SFS.1-CVB3ΔVP0-IRES-Venus#9 shows an apparently normal male karyotype (46,XY) after transfection with KAO717-pPB-hCMV1-CVB3ΔVP0-IRES-Venus. (**h**) Dose-dependent CVB3ΔVP0 expression in dependency of doxycycline concentrations. SFS.1-CVB3ΔVP0-IRES-Venus cells were treated for three days with increasing doxycycline concentrations and then stained with an antibody targeting CVB3-VP1, the secondary antibody was fluorescently labelled.

For many RNA-viral diseases, including SARS*-*CoV*-*2, Coxsackie and other RNA-virus infections, there are still no effective vaccines and/or treatments established^1^. Thus, improving diagnosis and treatment of viral infections is highly relevant in medical research. To conduct efficient research, valid disease models closely mimicking the pathology in humans are indispensable. So far, three approaches have been used to study viral infections: Via samples from humans (1), from animal models (2), and from human cell lines that were infected with virus particles in vitro (3)^2–5^. The opportunity of obtaining samples from humans is often limited, as tissue samples can only be collected from patients as biopsies or from the deceased. Therefore, a model system using human tissue, which is more easily accessible, is highly desirable and would solve that problem. With the development of (induced) pluripotent stem cells that virtually can proliferate indefinitely and potentially can differentiate into any kind of tissue, a high number of organ-specific cells can be obtained. However, to date, in vitro experiments were done by using infectious virus particles that require an appropriately equipped laboratory (often biosafety level 2 or higher) and that are potentially harmful for the experimenters and limit the possibility of standard laboratories allowed to conduct drug screening. To counter this limitation and enable research for normally equipped biosafety level 1 laboratories, we developed a human induced pluripotent stem cell (hiPSC) line that can express viral genes, but does not create infectious virus particles. As a model system for RNA viruses, we used the well-studied Coxsackie-virus B3 (CVB3) of the RNA-enteroviruses as infecting reagent. CVB3 was shown to cause myocarditis, meningoencephalitis, insulitis, diarrhea and insulin-dependent type 1 diabetes^6^. We stably transfected hiPSCs with a construct of the CVB3 virus genome that carries two mutations in the part of the virus genome encoding the viral capsid proteins (Fig. 1a). The CVB3-genome, including the two mutations henceforth called CVB3ΔVP0, prevents competent viral capsid formation, rendering the system non-infectious and thus makes its downgrade from biosafety level 2 to 1 possible^7^. This approach represents a huge advantage for experimenters and allows most labs highly controlled work on RNA-viruses. In addition, the construct has a doxycycline-dependent Tet-on promoter and a fluorescent Venus reporter, which enable the duration and degree of viral infection to be controlled and monitored by adjusting the time of application and the concentration of doxycycline administration (Fig. 1b).

For local and selective viral induction in some selected cells to mimic natural infection patterns, we aimed using caged-doxycycline. Cambridge et al*.* have principally demonstrated that localized gene expression using caged-doxycycline was possible, however in a very simple cell system^8^. Locally controlled de-caging leads to confined regions in which the virus is expressed. This procedure can be used to mimic typical infection patterns, which are usually observed in e.g. enteroviral myocarditis of humans and mice^9^.

## Results

### The generation of a CVB3DVP0-expressing hiPS-cell line

For the generation of a fully controllable, CVB3-expressing iPS-cell line, a triple-vector system was stably transfected into wildtype human iPS-cells named SFS.1. An overview of the functional triple-vector system for the generation of an inducible, CVB3ΔVP0 expressing iPSC-line is shown in Fig. 1. Further details of the cell-line generation are given in the online methods. The main vector in this transfection system, KAO717-pPB-hCMV1-IRES-Venus, is a well-established stem-cell Tet-On transfection vector^10–12^. The DNA of CVB3ΔVP0 was cloned into KAO717-pPB-hCMV1-IRES-Venus by extracting CVB3∆VP0 from a synthetic CVB3∆VP0-PUC53 using the restriction enzyme EcoR1 and then re-inserting it into KAO717-pPB-hCMV1-IRES-Venus within its EcoR1 cutting site. Expression is controlled by a CMV_min_ promoter and the co-expression of the Venus marker is ensured by a linked IRES2-sequence. The newly generated construct was named KAO717-pPB-hCMV1-CVB3ΔVP0-IRES-Venus (Fig. 1a). The vector KA0637-pPgCAG-rtTAM2-IN encodes the necessary rTA-trans-activator to enable doxycycline-inducible CVB3ΔVP0 expression. Furthermore, KA0637-pPgCAG-rtTAM2-IN encodes a G418 resistance allowing for positive selection after lipofection. In order to generate an effective cell-line it is important that KA0637-pPgCAG-rtTAM2-IN and KAO717-pPB-hCMV1-CVB3ΔVP0-IRES-Venus are inserted together into the genome of an iPS-cell. The co-transfected vector PB200PA-1 encodes the PiggyBac transposase that is essential for the stable random insertion of KA0637-pPgCAG-rtTAM2-IN and KAO717-pPB-hCMV1-CVB3ΔVP0-IRES-Venus into the iPS-cell genome. This vector requires only transient expression in the early phase after lipofection. In this study, lipofection was optimized for huge vector constructs allowing for transfer of even larger RNA-virus genomes like CVB3ΔVP0 (Fig. 1c, d). Positively transfected clones expressing CVB3ΔVP0 were picked and purified to generate a homogenously CVB3ΔVP0 expressing cell-line (Fig. 1e, f). The clones #8 and #9 were picked for further investigation. Karyotyping of SFS.1-CVB3ΔVP0-IRES-Venus#9 verified the male genotype of the generated cell line and the integrity of the cell´s chromosomes (Fig. 1g). Karyotyping was performed using standard GTG banding procedures.

A central factor for an effective viral model system is a tight control over viral gene expression. The amount of produced virus proteins is directly related to the virus load and allows to model different scenarios. In order to test our system for that applicability, different doxycycline concentrations were applied to SFS.1-CVB3ΔVP0-IRES-Venus#9 cells for three days. An immune staining that made the viral capsid protein CVB3-VP1 visible showed that the fluorescence intensity increased as a linear function of the doxycycline concentrations between 0.25 and 2.0 µg/ml. This is a clear indication of a controllable dose-dependent CVB3ΔVP0 expression.

The dose dependence of CVB3ΔVP0 expression on doxycycline offers the possibility to model different virus loads reflecting different phases of infection (Fig. 1h). This is important as in persistent CVB3 infections ongoing low levels of viral RNA are observed whereas in acute CVB3 infections we note high amounts of viral RNA especially in cardiomyocytes^9^. As CVB3 infections often target the human heart with acute and chronic outcomes, SFS.1-CVB3ΔVP0-IRES-Venus was used to differentiate human cardiomyocytes to study the effects of CVB3 expression in more detail.

### Expression of CVB3ΔVP0 in differentiated cardiomyocytes

Next, we tested, whether the expression of CVB3ΔVP0 is still possible after differentiation of hiPSC. Cellular differentiation processes potentially can silence or shut off chromosomal regions and inactivate the stable transfected construct unexpectedly. We chose to differentiate hiPSC into cardiomyocytes, since cardiac cells are a main target for RNA viruses like CVB3 and SARS*-*CoV*-*2 viruses in human patients leading to severe outcomes as cardiac death. For differentiation, we used a recently established protocol in which the Wnt and BMP signaling are activated on day 0 of differentiation followed by a selective Wnt shutdown at day 2 and day 3 of differentiation leading to a high amount of ventricular-like cells (~ 90% efficiency)^13^.

**Figure 2:**
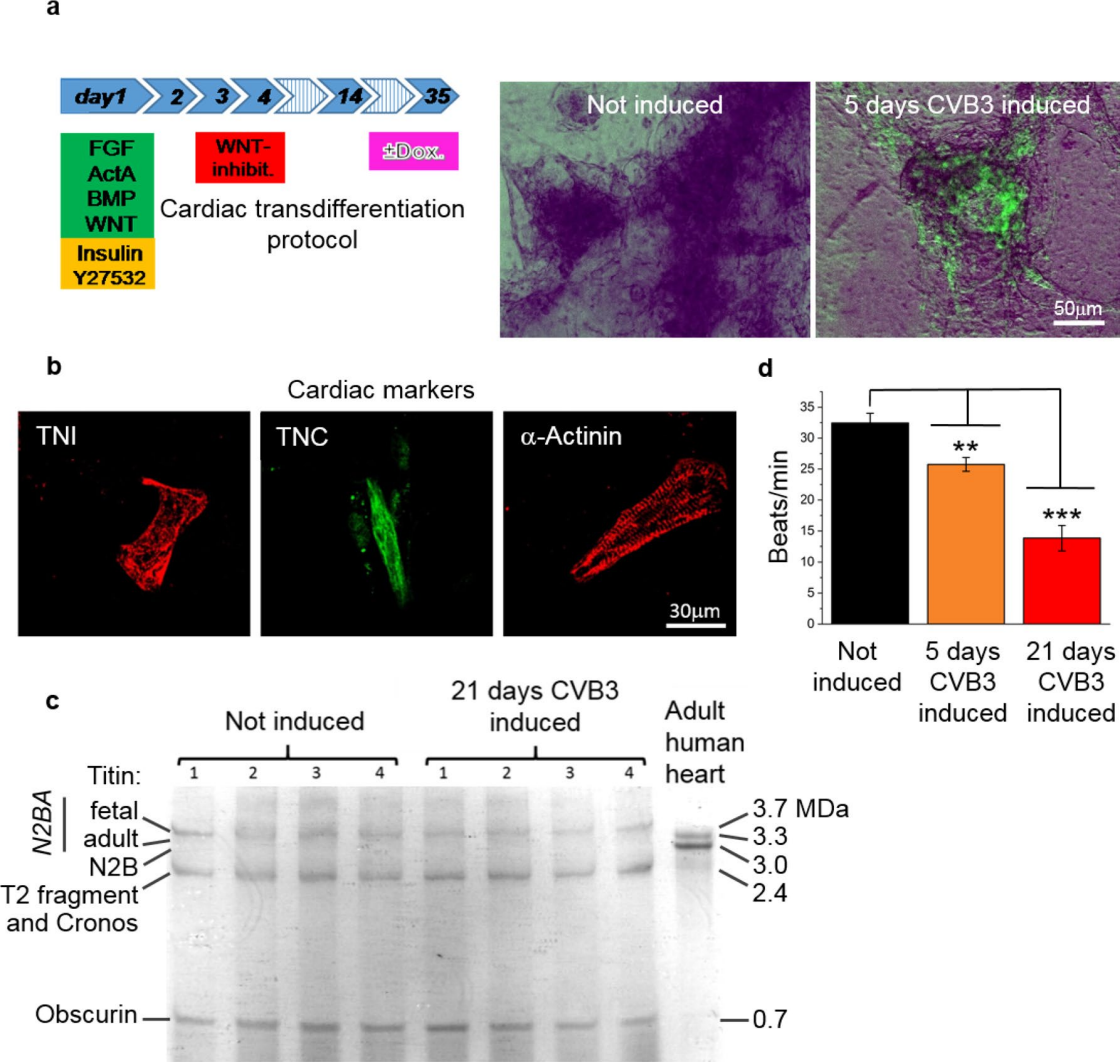
Expression of CVB3ΔVP0-IRES-Venus after cardiac differentiation. (**a**) Differentiation protocol and life cell images of differentiated cardiac cells at day 15, derived from SFS.1-CVB3ΔVP0-IRES-Venus#9. Schematic differentiation protocol used to differentiate SFS.1-CVB3ΔVP0-IRES-Venus#9 into functional cardiomyocytes. Left picture: SFS.1-CVB3ΔVP0-IRES-Venus#9-derived cardiomyocytes without doxycycline treatment. Right picture: SFS.1-CVB3ΔVP0-IRES-Venus#9-derived cardiomyocytes treated with 2 μg/ml doxycycline for 5 days. Cardiomyocytes treated with doxycycline show strongly visible Venus marker indicating CVB3ΔVP0 expression. (**b**) Immunostaining of cardiac markers TNI, TNC and α-actinin proves the cardiac phenotype of SFS.1-CVB3ΔVP0-IRES-Venus derived cardiomyocytes. (**c**) Coomassie staining of a 1.8% polyacrylamide/1% agarose gel resolving the expression of the fetal Titin isoform N2BA and the giant sarcomeric signaling protein obscurin in SFS.1-CVB3ΔVP0-IRES-Venus#9-derived cardiomyocytes. (**d**) Expression of CVB3ΔVP0 lowers the beating rate of SFS.1-CVB3ΔVP0-IRES-Venus#9-derived cardiomyocytes over time. Error bars represent the ± SEM of 20 averaged independent cellular samples per condition (n = 20).

The generated cardiomyocytes grow as monolayer and are spontaneously active (Supplementary Video 1), facilitating physiological measurements and allowing comparisons between virus-expressing and non-virus-expressing cells. Differentiated cardiomyocytes are capable of expressing the transfected CVB3ΔVP0-IRES-Venus construct after 5 days doxycycline treatment as live cell fluorescence imaging of Venus in differentiated cardiomyocytes clearly demonstrates (Fig. 2a). Immunostaining visualizing typical cardiac markers cardiac troponin I (TNI), cardiac troponin T (TNT) and α-actinin, prove the cardiac phenotype of the differentiated cells (Fig. 2b). Furthermore, analysis of large sarcomeric proteins with loose gel electrophoresis showed the presence of fetal titin N2BA and the sarcomeric signaling protein obscurin, demonstrating the exceptional good quality of the iPS-cell derived cardiac myocytes (Fig. 2c). In order to test for possible effects of the expression of CVB3ΔVP0-IRES-Venus on the human iPS-cell derived cardiomyocytes, the expression of the viral genes was induced with 2 μg/ml doxycycline for 5 days and for 21 days. As a readout for putative effects, the beating frequencies of the induced cells were compared to non-induced control cells. We found significant changes in the beating rates: Non-induced, four weeks maturated, cardiomyocytes showed a regular beating at a rate of approximately 35 ± 1.56 (mean ± SEM) beats per minute at 25 °C. iPS-cell derived SFS.1-CVB3ΔVP0-IRES-Venus#9 cardiomyocytes of the same age, which were treated with doxycycline for 5 days and 21 days showed significantly less contractions per minute. Cells induced for 5 days contracted 26 ± 1.12 (mean ± SEM) times per minute, while cells induced for 21 days contracted 14 ± 2.04 (mean ± SEM) times per minute on average (Fig. 2d). Furthermore, beating of cells induced for 21 days becomes irregular and uncontrolled (Supplementary video 2). These observations thereby cannot be explained with induced apoptosis by doxycycline as the performed life/dead staining of SFS.1CVB3DVP0-IRES-Venus#9 and SFS.1wt indicates (supplementary Fig. 1c). The expression of CVB3ΔVP0 thus exerts adverse effects on cell physiology, as expected for a well-defined disease model. Together with the previously described dose-dependent induction, the produced CVB3ΔVP0-IRES-Venus cell-line is functional and inherits great potential to study pathophysiological effects of CVB3 in a highly controlled, scalable human cell model system. Moreover, the expression of CVB3 can be controlled and fine-tuned, resulting in a population of cells, which are exposed to very similar pathophysiological conditions. This will enhance homogeneity/reduce variability and thus will yield clearer results.

To further confirm the functionality of CVB3ΔVP0 induction in the generated cell system, a physiological characterization of differentiated cardiomyocytes concerning mitochondrial ROS production and alterations in electrochemical signal generation was performed. Previous studies of CVB3 infections already demonstrated significant impact of CVB3 on ROS production and changes in signal transduction in cardiac cells^14,15^. The expression of CVB3ΔVP0 was induced in differentiated cardiomyocytes for 21 days. The cells were stained with MitoTrackerCMXRos and analyzed for enhanced ROS production, as indicated by elevated fluorescence intensities in fluorescence microscopy. The fluorescence intensities in the cytosol and in mitochondria of 21 days induced cells and non-induced control cells were compared and statistically analyzed.

**Figure 3:**
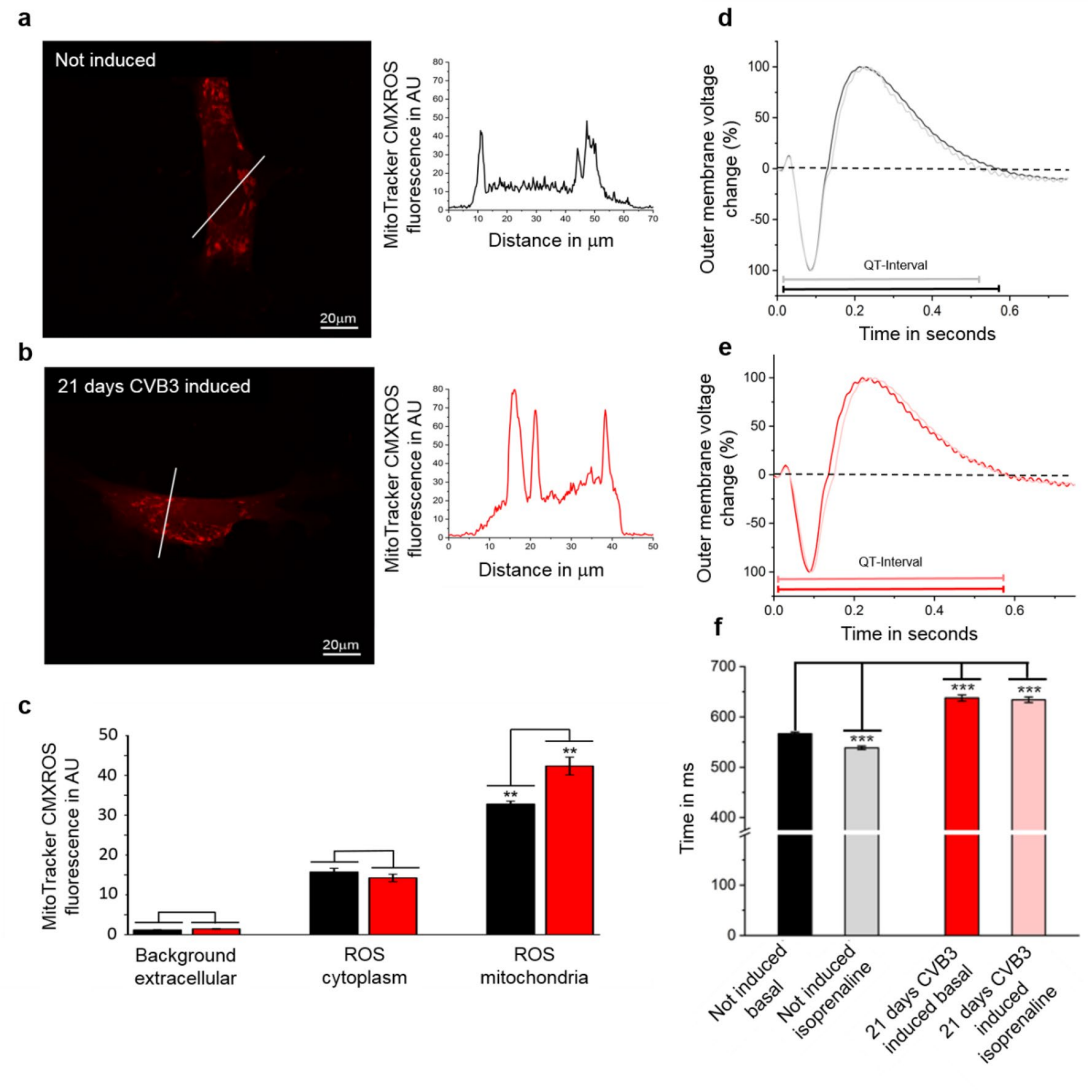
The expression of CVB3ΔVP0 elevates mitochondrial ROS production and changes electrochemical cell signaling. (**a**) MitoTrackerCMXRos staining of a non-induced SFS.1-CVB3ΔVP0-IRES-Venus#9 (control) cell analyzed by linescanning for fluorescence intensity indicating the relative ROS content in mitochondria. (**b**) MitoTrackerCMXRos staining of an induced SFS.1-CVB3ΔVP0-IRES-Venus#9 cell (21 d) analyzed by linescanning for fluorescence intensity indicating the relative ROS content in mitochondria. (**c**) Statistical analysis of MitoTrackerCMXRos fluorescence intensities of 21 days SFS.1-CVB3ΔVP0-IRES-Venus#9 induced cardiomyocytes and non-induced SFS.1-CVB3ΔVP0-IRES-Venus#9 control cells in comparison (n = 8, ** indicates *p* < 0.01). (**d**) Exemplary multi-electrode array recording of non-CVB3ΔVP0 induced SFS.1-CVB3ΔVP0-IRES-Venus#9 control cells under basal conditions (dark grey) and exposed to 10 μM isoprenaline (light grey). (**e**) Exemplary multi-electrode array recording of 21 days CVB3ΔVP0 induced SFS.1-CVB3ΔVP0-IRES-Venus#9 cells under basal conditions (dark red) and in presence of 10 μM isoprenaline (light red). (**f**) Statistical analysis of equivalent QT-Intervals of multi-electrode array recordings of 21 days CVB3ΔVP0 induced SFS.1-CVB3ΔVP0-IRES-Venus#9-derived cardiomyocytes and non CVB3DVP0-induced control cells in comparison under basal conditions and with 10 μM isoprenaline applied (n = 5, ***p < 0.001).

Fluorescence microscopy of MitoTrackerCMXRos was performed with a 63 × oil immersion objective under constant conditions to ensure comparable fluorescence intensities in the generated samples of SFS.1-CVB3ΔVP0-IRES-Venus#9 and SFS.1-CVB3ΔVP0-IRES-Venus#8. For fluorescence intensity recording, a linescan was performed documenting the fluorescence intensities of MitoTrackerCMXRos in the mitochondria, the cytoplasm and the extracellular space (Fig. 3a–c; supplementary Fig. 1d–f). The fluorescence of MitoTrackerCMXRos was not altered in the cytoplasm and the extracellular space of cardiomyocytes induced for 21 days compared to non-induced cells. Within the mitochondria, MitoTrackerCMXRos fluorescence intensity was elevated in 21 days CVB3ΔVP0 induced cardiomyocytes compared to the non-induced control indicating an elevated ROS production caused by CVB3ΔVP0 expression. Additionally, a multi-electrode array (MEA) study was performed on SFS.1-CVB3ΔVP0-IRES-Venus#9-derived cardiomyocytes to verify the impact of CVB3ΔVP0 expression on cellular signal generation. Hereby, the outer membrane voltage alterations of cells within a cellular signal generation process, like field potentials, are monitored, which can give information about the cell´s physiological activity. As indicator for electrochemical signal alterations related to beating, the QT-Interval of 21 days CVB3ΔVP0 induced and non-induced cardiomyocytes was analyzed under basal conditions and under β-adrenergic stimulation with 10 μM isoprenaline. Exemplary MEA measurements of 21 days CVB3ΔVP0 induced and non-induced cardiomyocytes are shown in Fig. 3d,e. Statistical analysis of the equivalent QT-intervals of induced and non-induced cardiomyocytes under basal conditions shows a significant equivalent QT-elongation in 21 days induced cells compared to the control. Under β-adrenergic stimulation with isoprenaline non-induced cells react with an expected equivalent QT-shortening compared to the basal condition (Fig. 3d,f), whereas induced cells do not show any reaction to β-adrenergic stimulation (Fig. 3e,f). Equivalent QT-intervals stay continuously elongated in presence of isoprenaline indicating a desensitization of induced CVB3ΔVP0 cardiomyocytes against β-adrenergic agonists.

The controlled expression of CVB3ΔVP0 does not only change the mitochondrial structure and electrochemical activity of cells, but also disrupts membrane integrity and leads to alterations in the cardiomyocyte phenotype. To image the three-dimensional structure of iPS-cell derived cardiomyocytes, 3-D holographic imaging (Nanolive) was performed visualizing cell structures.

**Figure 4:**
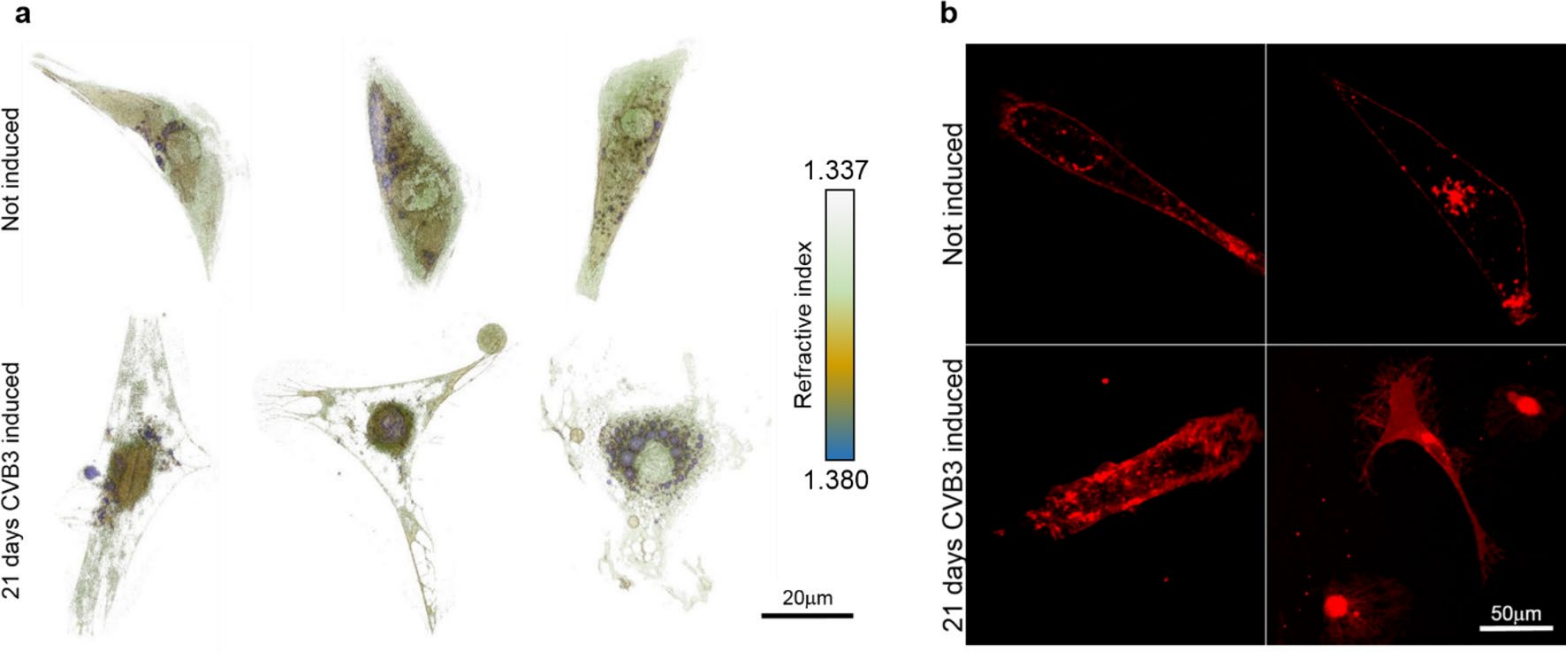
CVB3ΔVP0 disintegrates cardiomyocyte inner membrane structures and leads to enhanced vacuolization. (**a**) Holographic 3D scan (Nanolive) of SFS.1-CVB3ΔVP0-IRES-Venus#9-derived cardiomyocytes under basal conditions and after 21 days of CVB3ΔVP0 expression. Digital staining of cellular structures marks vacuoles (blue), intracellular membrane structures (orange) and cytoplasmatic regions (green). (**b**) Membrane staining of living SFS.1-CVB3ΔVP0-IRES-Venus#9-derived cardiomyocytes with CellMask deep red at a concentration of 2.5 μg/ml.

The 3D scan of living iPS-cell derived cardiomyocytes expressing CVB3ΔVP0 for 21 days revealed drastic changes in overall cell morphology and internal structures while cardiomyocytes without CVB3ΔVP0 expression appear healthy and vital with a high density of intracellular membrane structures as mitochondria and SR and a well-defined cell membrane border. Intracellular membrane structures are partially degraded and the cell membrane borders seem frayed. In addition, CVB3ΔVP0-expressing cardiomyocytes show a high density of intracellular vacuoles marked in blue, compared to the non-induced cardiomyocytes (Fig. 4a, supplementary Fig. 2a). These findings are supported by membrane staining with CellMask deep red in living cardiomyocytes. Non-induced cardiomyocytes show well-defined and clear cell membrane borders as well as a structured intracellular organization of membranes of organelles. After 21 days of CVB3ΔVP0 induction the intracellular membrane organization seems disturbed and degraded. The staining of many spherical structures within the induced cells is reminiscent of vacuole-like structures, which corresponds to the observations made with the holographic 3D scan. Furthermore, the cell membrane borders are not well-defined anymore and appear frayed as in the holographic images (Fig. 4b, supplementary Fig. 2b).

In summary, here we showed the functionality and applicability of CVB3ΔVP0 to mimic RNA-viral infections under highly controlled and safe conditions. We demonstrated the CVB3ΔVP0 impact on cardiomyocyte function and revealed a disintegration of several cellular organelles as a result of virus infection. However, it has to be mentioned that native viral infection patterns appear focal. Therefore, in a further line of experiments, we tested the SFS.1-CVB3ΔVP0-IRES-Venus cell-model for its feasibility to simulate a patterned virus induction to simulate infection foci.

### Photoactive caged-doxycycline enables patterned CVB3ΔVP0 expression

**Figure 5:**
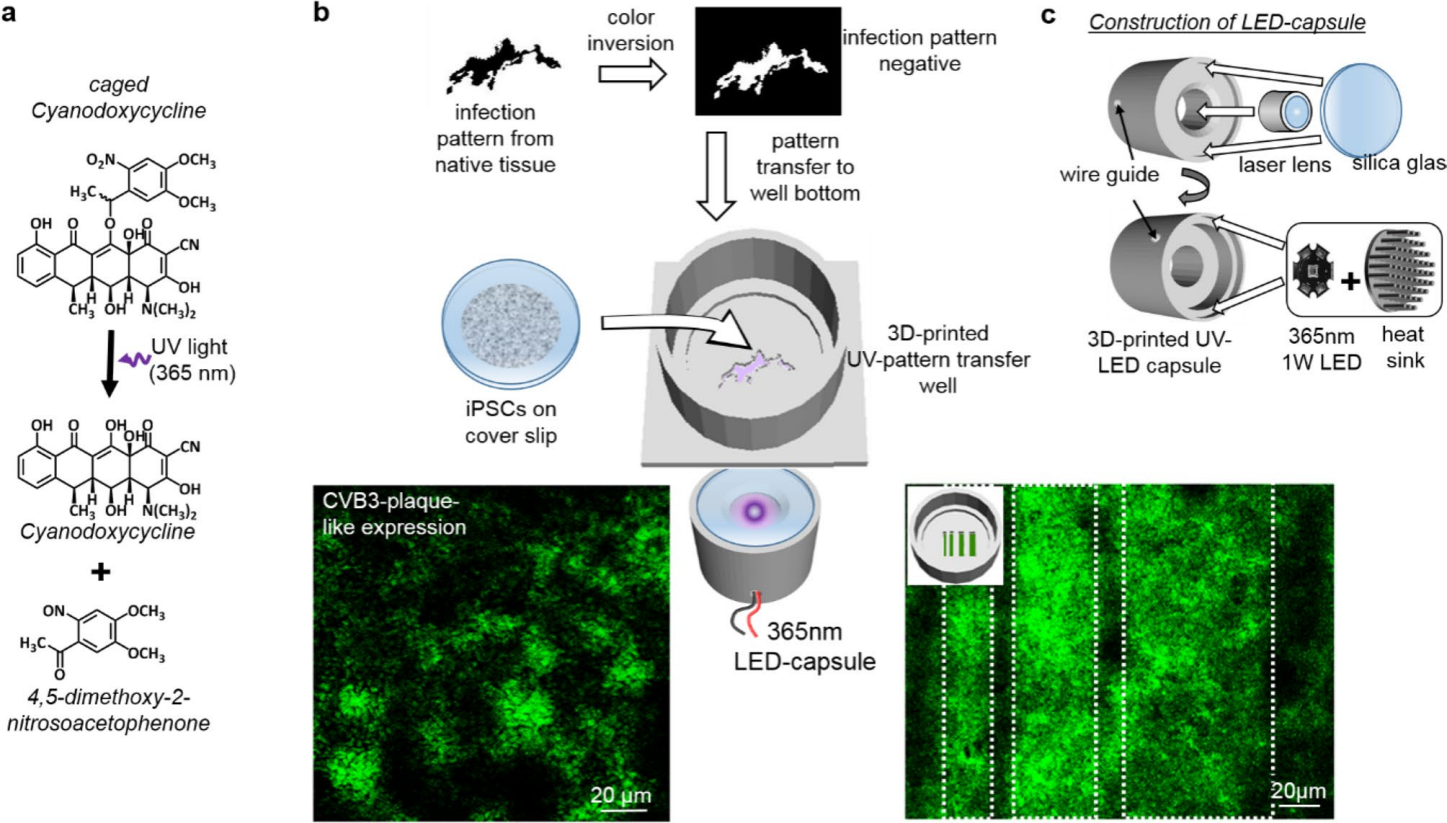
Schematic overview of experimental flow for controlled localized viral induction on 2D tissue layer using caged-doxycycline. (**a**) Upon UV-irradiation, caged-doxycycline breaks down into cyanodoxycycline and the non-toxic side product 4,5-dimethoxy-2-nitrosoacetophenone. (**b**) Schematic experimental setup. Patterns of CVB3 infections can mimic published or self-generated shapes and have to be inverted by a graphical software. This negative can be used to adapt the bottom of 3D-printed wells. Alternative patterns like stripes of variable breath may be used as well. SFS.1-CVB3ΔVP0-IRES-Venus#9 were plated on glass cover slips and transferred in these 3D-printed wells. Cells were incubated with caged-doxycycline first and then UV-radiated from the bottom at specific locations. Initiation of viral transcription according to chosen patterns can be observed by fluorescence of the Venus marker after 24 h. (**c**) Construction of a versatile waterproof UV-illumination LED-capsule: Light from a 1 W-365 nm-LED is focused by standard laser lens to illuminate the cells from the bottom.

As RNA-virus infections in the human patient do not occur homogenously throughout an infected tissue, but appear in so called plaques, we asked whether a localized induction of CVB3ΔVP0 in SFS.1-CVB3ΔVP0-IRES-Venus#9 would allow to simulate CVB3 plaques in a petri dish. For this purpose, a new derivative of doxycycline, caged-doxycycline (c-Dox), was synthesized and applied to the cell system. Caged-doxycycline is inactive until UV-light breaks it down into active cyano-doxycycline and a non-toxic residue 4,5-dimethoxy-2-nitrosoacetophenone (Fig. 5a). UV-irradiation and c-dox concentrations below 200 μg/ml thereby have no significant effect on cell survival (supplementary Fig. 1b).

SFS.1-CVB3ΔVP0-IRES-Venus#9 cells were plated on 12 mm glass coverslips, inserted into newly designed and 3D printed wells with defined cavities on the bottom (Fig. 5b). C-Dox was applied to the cells for a short incubation time, then washed away. Via UV-irradiation, coming from the well bottom from a newly designed LED-Capsule (Fig. 5c), c-dox is broken down into active cyanodoxycycline, initiating the CVB3ΔVP0 expression in the illuminated cell layer. This approach creates the possibility to induce a very localized and controlled expression of the virus in cells from the SFS.1-CVB3VP0-IRES-Venus cell line in order to simulate the effects of CVB3 on local cell clusters (supplementary Fig. 3). With this tool in hand, it is now possible to observe changes in excitation spreading and contraction propagation if a signal passes CVB3 expressing cell clusters embedded in a non-induced control cell layer. As CVB3 is known to modulate cardiac ion-channels in membrane localization and function, the effects of CVB3-infected cell clusters in a non-infected monolayer are a valuable object for quantitative and qualitative analysis^16,17^. Moreover, completely new experimental setups can be designed and the pathophysiology of RNA-virus infections can be studied in more detail than ever before, especially when it comes to high-speed life cell imaging or in the use of voltage-dependent dyes.

## Discussion

The newly produced human cell-line SFS.1-CVB3ΔVP0-IRES-Venus enables the investigation of RNA-virus-induced-effects in an entirely controllable, experimental setup. The complete genetic information of a non-infectious, but replicative RNA-virus strain (CVB3ΔVP0), linked to a Tet-On system for doxycycline dependent transcription, was stably transfected into the human iPS-cell line SFS.1. This setup provides the basis for non-infective and tightly controllable experiments with the RNA-virus CVB3. It is now possible to design experiments to determine the effects of different viral loads in various induction states under numerous aspects. As this RNA virus model system is human iPS-cell based, it is moreover possible to expand observations of RNA-virus effects to multiple other cell types in addition to cardiomyocytes. In the case of CVB3, the virus infects patient cells of the pancreas, the central nervous system and the respiratory tract. SFS.1-CVB3ΔVP0-IRES-Venus represents a basis to study viral infections in the desired cell types via transductions^6,18,19^.

Changes in the beating frequency of CVB3ΔVP0 induced, iPS-cell derived cardiomyocytes at different time points proved the principle and the functionality of the designed model.

Physiological characterization of differentiated CVB3ΔVP0-expressing cardiomyocytes showed a significant increase of ROS production in the mitochondria. This observation correlates with findings of previous studies in infected mouse tissues in which elevated ROS levels are observed as well^14,15,20^. CVB3ΔVP0 expression also changed the electrical activity of the iPS-cell derived cardiomyocytes on the multi-electrode array. CVB3 induced cells showed an elongated QT-interval compared to the non-induced control cells. After supplementation of isoprenaline, non-induced cells reacted with a QT-shortening while induced cells showed no significant changes in the QT-interval. It might be, that the expression of CVB3ΔVP0 leads to a desensitization of cardiac cells towards β-adrenergic stimulation, either via proteolytic degradation of cAMP binding-sites of acceptor proteins or via continuously elevated cAMP-levels, which were found in infected mouse cardiomyocytes^15^. Continuously elevated cAMP-levels in mouse cardiomyocytes could explain the observed desensitization against β-adrenergic stimulation, which is a cAMP-activating process.

The expression of CVB3ΔVP0 in human iPS-cell derived cardiomyocytes also led to drastic changes in cell morphology and membrane structures. While non-induced cells showed a healthy phenotype with intact cell membrane and intracellular membrane structures, virus-induced cells appeared dysregulated in their membrane integrity. Intracellular membrane structures were degraded as well and many cells showed an increase of vacuolic structures. These observations are consistent with the results of previous studies, which postulated that the viroporin 2B of CVB3 is capable of disrupting membrane structures^21–23^. This disruption is accompanied by a degradation of mitochondria and the golgi apparatus and an increase in intracellular vesicles and vacuoles, as two independent imaging methods confirmed. Overall, the expression of CVB3ΔVP0 seems functional and well controllable in the presented system and gives the user the ability to study the effects of CVB3 in a controlled and time-dependent environment without the risk of infection. Moreover, the use of UV-activated doxycycline additionally offers for the first time the possibility of localized virus expression with a confinement of several hundred µm. This will lead to even more complex and detailed experimental designs in the future in which the influence of modeled viral plaques and infected compartments of the heart and other tissues can be assessed. The here presented system provides a novel approach to apply gene editing, in a highly controlled, versatile expression system of a pathogen in human iPS generated cell lines.

## Supplementary information

Supplementary video 1.

Supplementary video 2.

Supplementary information.
