# Supplementary material for: The first versatile human iPSC-based model of ectopic virus induction allows new insights in RNA-virus disease
Source: Sci Rep. 2020 Oct 8;10:16804. doi: 10.1038/s41598-020-72966-9 (PMC7546621; doi:10.1038/s41598-020-72966-9)
Supplement: Supplementary file 3 — Supplementary information. [file 41598_2020_72966_MOESM3_ESM.docx]

**Online Methods**

**iPS-cell culture**

Sendai Foreskin 1 (SFS.1) human iPS cells^1^ were incubated at 37°C and 5% CO_2_. SFS.1 were cultured as described by Frank et al.^2^. Briefly summarized, SFS.1 were cultured in FTDA medium (DMEM/F12) (Invitrogen #21331020), 5 µg/ml ITS (Becton Dickinson #354350), 0.1% human serum albumin (Biological Industries #05-720-1B), 1X CD Lipid Concentrate (Invitrogen #1905031), 1X Penicillin/ Streptomycin/ Glutamine (Life Technologies #10378016), 10 ng/ml FGF2 (PeproTech #100-18B), 5 ng/ml Activin A (eBioscience #34-8993-85), 0.4 µg/ml TGFβ1 (eBioscience #34-8348-82), and 50 nM Dorsomorphin (Santa Cruz #sc-200689)). Medium was changed daily. After 3 to 5 days of culture when cells reached full confluence, cells were passaged by washing once with PBS and incubating with Accutase^®^ solution (Sigma #A6964) supplemented with 10 µM Y-27632 (Abcam Biochemicals # ab120129) for approximately 10 minutes at 37 °C. The reaction was stopped by adding FTDA medium supplemented with 10 µM Y-27632. The detached cells were centrifuged for 2 min at 200 x g and re-suspended in FTDA medium supplemented with 10 µM Y-27632. For further culture, approximately 600,000 cells were distributed onto each 6-well that were previously coated with 1:75 diluted Matrigel^®^ (Becton Dickinson #354263). After one day, medium was changed into FTDA medium without Y-27632.

**Generation of a transgenic Coxsackievirus-inducible human iPS-cell line**

The described three vector system was used to produce the inducible, CVB3∆VP0 expressing, cell line SFS.1-CVB3ΔVP0-IRES-Venus. The vectors were aimed to be transfected into the iPS-cell line SFS.1wt via FuGeneHD-lipofection in a vector ratio 1:3:15 (PB200PA-1: KA0637-pPgCAG-rtTAM2-IN: KAO717-pPB-hCMV1-CVB3ΔVP0-IRES-Venus)^3^. FugeneHD is recommended for huge vector constructs like KAO717-pPB-hCMV1-CVB3ΔVP0-IRES-Venus with a size of 14220bp, providing a higher transfection efficiency compared to FuGene6. Of note, first transfection attempts with FuGeneHD with the companies recommended cell counts and plasmid concentrations were without success.

To ensure that the transfection conditions with FuGeneHD are suitable and that FuGeneHD is the optimal choice for this transfection, intense troubleshooting was performed. In a first set of experiments the transfection efficiency of FuGeneHD was compared to FuGene6 with a standard cell count of 150.000 cells on a 6 well format. The vector ratio was kept unchanged at 1:3:15. In FACS analysis FuGene6 showed a two times higher transfection efficiency with pcDNA3-CrispR-GFP, which was used as test vector with a size of approximately 13kb (Fig. 1c and 1d). Further transfection experiments were then performed with FuGene6. Follow up adjustments to the transfection procedure were done on a 24-well format. As the cell count and vector ratio strongly impact transfection efficiency these two parameters were varied in a further experiment. The highest transfection efficiency with FuGene6 was observed with a cell count of 20.000 cells/24 well and a vector ratio of 1:3:15 with a transfection efficiency of 28.77%. Lower or higher cell densities of 10.000-50.000 cells/24well and a changed vector ratio of 1:3:10 did not show improved transfection efficiencies, so the transfection efficiency of 28.77% was taken as final working condition.

Further transfections were then performed using the desired vector KAO717-pPB-hCMV1-CVB3ΔVP0-IRES-Venus instead of pcDNA3-CrispR-GFP. Experiments were done under the optimal validated conditions. 24 hours after transfection with FuGene6 the selection of positively transfected cells was started. In a first selection step the cells were kept in FTDA medium containing 50 μg/ml G418 for 7 days. Cells positively transfected with the vector KA0637-pPgCAG-rtTAM2-IN, are expected to be resistant to G418 and stay vital, while cells not containing this vector should undergo apoptosis. In a second selection step G418 was removed from the FTDA medium and replaced with doxycycline at a concentration of 1 μg/ml. This step activates the rTA-trans-activator and induces the expression of the transfected KAO717-pPB-hCMV1-CVB3ΔVP0-IRES-Venus vector construct, if transfection was successful (Fig. 1b)^3^. Cells were checked daily for green Venus-marker fluorescence indicating presence of all three vectors within one cell, which was expected to have already grown into a colony, days after the initial transfection procedure. Astonishingly, even after an abundant amount of transfection experiments no Venus-positive colony was observed.

From the data of the transfection procedure optimization, the transfection with KAO717-pPB-hCMV1-CVB3ΔVP0-IRES-Venus should work without problems, so there had to be a specific problem with this vector insert in itself. We suggested that the screening with doxycycline may represent a problem. The doxycycline dependent activation of rTA induces the expression of CVB3ΔVP0 along with the Venus marker. But if CVB3ΔVP0 is transcribed into the transfected cells, viral effects can impact cell growth and proliferation. Indeed, CVB3, like many other viruses is known to have significant influence on cell physiology and is able to shut down cellular translation^4,5^. If this is the case in our system as well, the screening of a transgenic CVB3 expressing cell-line would be rendered impossible. Positively transfected cells would die or stop growing before a detectable Venus signal could be observed for successful colony picking. Even a follow up expansion of these picked cells would be questionable due to long lasting viral effects remaining in the cells, even after doxycycline removal. In the case of CVB3 infections it is also known that CVB3 encodes for two proteases, 2A and 3C, which are responsible for the majority of the observed effects on cell translation^6^. The blockage of proteolytic activity in the screening step was chosen to increase the chances of picking a functional cell line. For this purpose, complete Protease Inhibitor Cocktail Tablets in EASY packs (Roche) following the manufacturer’s recommendations were used, to block proteolytic activity possibly affecting the cells’ growth.

Indeed, with the protease inhibitor cocktail present in the medium, several Venus expressing colonies were observed three days after doxycycline application at first try (Fig. 1e). Of note, selective inhibition of viral protease 3C by medium supplementation with 0.5 μM Ag7088, did not result in fluorescent colonies. This indicates that inhibition of protease 3C alone is insufficient to allow for colony formation. Nine of these colonies were picked, expanded and observed over three passages. Two further picking and reseeding steps were performed to increase the purity of the clones (Fig. 1e). The two best growing clones, clone #8 and #9, were kept in culture and checked for their Venus expression homogeneity with FACS. For this purpose, cells were induced for 5 days with 1 μg/ml Doxycycline. The induced cells were dissociated into single cells using 1ml of 1 x Accutase, supplemented with 10 µM Y-27632 for 12 min. The dissociation reaction was stopped with 1 ml FTDA medium. The cells were analyzed for their Venus expression using the Beckman Coulter Gallios instrumentation. The FACS analysis showed complete Venus expression in both of the clones and proved their purity (Fig. 1f). The newly generated cell line was named SFS.1-CVB3ΔVP0-IRES-Venus. Karyotyping of SFS.1-CVB3ΔVP0-IRES-Venus verified the masculine genotype of the generated cell line and the integrity of the cell´s chromosomes (Fig. 1g). Karyotyping was performed using standard GTG banding procedures.

**Immunofluorescence staining and imaging**

Immunostaining was performed by fixing the cell samples in 4% PFA/PBS for 10 min and washed gently with PBS-T. Blocking was performed for 1h with 2% BSA, 2% glycine, 0,2% Triton-X in PBS-Tween (PBS-T). Cells were rinsed with PBS-T and incubated with the primary antibodies against the viral capsid protein CVB3-VP1, cardiac troponin I (TNI) (ab171650, Abcam), cardiac troponin T (TNT) (ab196683, Abcam), and α-actinin (A7811, Sigma Aldrich) at 4°C, overnight in 0.5% BSA, PBS-T in a ratio 1:500. Cells were subsequently washed with PBS-T three times and incubated with the secondary antibody (A7811 or SAB4600036 Sigma Aldrich) in 0.5% BSA, PBS-T in a ratio of 1:1000. Cells were mounted on glass and imaged with a confocal microscope (DMI4000 from Leica). For membrane imaging living cells were plated on matrigel and gelatin coated, glass bottom, 35 mm μ-dishes (#81158 Ibidi) in a density of 100.000 cells/dish and cultured over night at 37°C and 5% CO_2_. The next day the cells were rinsed two times with PBS and then supplemented with a staining solution containing 1ml DMEM with 1% FCS and 1% Glutamine and 0.5 μL CellMask™ deep red membrane stain (Invitrogen). The cells were incubated in the staining solution for 10min, then rinsed with PBS two times and imaged with DMEM with 1%FCS and 1% Glutamine without phenole red. The cells were imaged at 37°C and controlled 5% CO_2_ with excitation at 649 nm and emission at 666 nm. HyD's with GaASP photocathodes were used as detectors for dual color recording.

**MitoTracker™CMXRos staining and statistical analysis**

4 weeks matured IPSC derived cardiomyocytes, either non-induced or 21 days induced, were seeded on 12 mm glass coverslips in a density of 100.000 cells and cultured in KO-THAI medium with or without 2 μg/ml Doxycycline overnight. Culture medium was then removed and cells were washed 2 x with PBS. MitoTracker™CMXRos/ PBS solution was prepared with a MitoTracker™CMXRos concentration of 100 mM. PBS was removed from the samples and replaced by 0.5 ml MitoTracker™CMXRos solution for 15min. Subsequently, cells were washed 2 x with PBS, fixed with 4% PFA/PBS for 10min and mounted on glass with AquaPolymount. The fixed cells were imaged with the confocal microscope (DMI4000 from Leica). Line scanning analysis was performed with the ImageJ software.

**Multi-electrode measurement and QT-Interval analysis**

4 weeks matured IPSC derived cardiomyocytes, either non-induced or 21 days induced, were seeded onto MEA Chips 256-9wellMEA300/30iR-ITO-mq (Multichannel Systems) in a density of 100.000 cells/well. The seeded cells were kept at 37°C and 5% CO_2_ over night for descent attachment in KO-THAI medium + 10 µM Y-27632. The next day media was changed to KO-THAI without Y-27632. Cells were kept at 37°C and 5% CO_2_ for another 24h. For multi-electrode measurement the seeded 256-9wellMEA300/30iR-ITO-mq Chip was loaded into the USB-MEA256-System (Multichannel Systems). The stimulus generator STG4000 (Multichannel Systems) was attached to the USB-MEA256-System as well. After performance of a viability test to verify functionality and background noise of the MEA-Chips electrodes, the measurement of the seeded iPSC-derived cardiomyocytes started. First the cells were measured under basal conditions for 5 min. Then a constant depolarization stimulus of 1000 mV every 999ms was applied to the cells for dependable cellular activation. The cell signals were monitored for 20 min. Following 10^-6^ M isoprenaline was applied to the cells for β-adrenergic stimulation. The cells were monitored for another 20 min. The recorded cell signals were analyzed with the Cadrio2D+ software (Multichannel systems). Equivalent QT-intervals of induced and non-induced cardiomyocytes under basal condition and β-adrenergic stimulation were measured and averaged (10 signals/sample).

**Nanolive 3D-Scan**

Differentiated cardiomyocytes were seeded in a density of 100.000 cells onto matrigel and gelatin coated glass bottom 35mm μ-dishes (#81158 ibidi) and cultured over night at 37°C and 5% CO_2_. The following day the medium was changed to Dulbecco´s modified eagle´s medium without phenol-red, supplemented with 1% Glutamine and 1% FBS. Subsequently the seeded glass bottom dishes were transferred into the incubation chamber of the Nanolive™ 3D imaging system with controlled temperature and 5% CO_2_. With the 63x objective of the Nanolive scanner, cells were focused and scanned according to the manufacturer´s instructions. Contrasting and digital staining of the acquired pictures was achieved with the STEVE-Software (Nanolive™ CH GmbH).

**Differentiation of SFS.1-CVB3ΔVP0-IRES-VENUS into cardiomyocytes**

SFS.1-CVB3ΔVP0-IRES-Venus were detached as described before. After pelleting, the cells were re-suspended in differentiation medium consisting of KO-DMEM (Life Technolgies #10829018), 1X Penicillin/Streptomycin/ Glutamine, 5 µg/ml ITS, 10 µM Y-27632, 20 ng/ ml FGF2, 1 nM CHIR-99021 (Axon Medchem #Axon1386), and 0.25 to 2.0 nM BMP-4 (R&D # 314-BP-010) that has to be regularly tested after a few passages and were seeded on Matrigel^®^ coated wells at 500,000 cells/ 24-well. Medium was changed after 24 hours to TS-ASC medium (KO-DMEM, 5.5 mg/L Transferrin (Sigma #T8158-100MG), 6.75 µg/L Selenium (Sigma # S5261-10G), 1X Penicillin/ Streptomycin/ Glutamine, 250 µM ascorbate (Sigma # 49752-10G). Cells were incubated in this medium for 24 hours and changed into TS-ASC medium that was supplemented with 0.5 µM C59 (Tocris #5148). Wnt was inhibited for 48 hours while medium was exchanged once in between after 24 hours. Subsequently, cells were matured by incubating the cells in TS-ASC without cofactors until they beat autonomously, usually around day 8 after differentiation initiation (Fig. 2a). Cells were washed once with PBS. Non-beating cells were mechanically detached with a pipette tip and removed. The remaining beating cells were incubated in TrypLE Select (1X) (Life Technologies # 12563011) with 10 µM Y-27632 for 10 min at 37 °C. Cells were then seeded in KO-THAI medium (KO-DMEM, 1X Penicillin/ Streptomycin/ Glutamine, 0.2% human serum albumin, 250 µM ascorbate, 5 µg/ml ITS, and 0,004% (v/v) Thioglycerol) at a 1:3 to 1:4 ratio on wells that were previously coated with 1:75 diluted Matrigel and 0.2% gelatin in a ratio of 1:1. Medium was changed into KO-THAI medium without Y-27632 after 24 hours and afterwards was changed every 2-3 days. Cells were passaged when confluence was reached with TrypLE Select (1X) as described above.

**Proteomic analysis of iPS-cell derived cardiomyocytes:**

Differentiated, 35 days old, cardiomyocytes of the cell line SFS.1-CVB3ΔVP0-IRES-Venus#9 were washed once with PBS. After removal of the PBS, the cells were mechanically detached with a 10 μL pipette tip and transferred to a 1.5 ml eppendorff tube. The cells were centrifuged at 200xg for 3 min and the supernatant was removed carefully. The isolated cell pellets were lysed under the use of urea-buffer consisting of 30 mM trisbase, 2 M thiourea, 7 M urea and two different kinds of glass beads with a size of 0.25-0.5 mm and 1.25-1.65 mm. The cell lysis was supported by the repeated use of a VialTweeter (Hielscher Ultrasonics GmbH, Germany). Subsequently after cell lysis, the protein concentration of the generated samples was determined via Bradford assay. Prior to digestion samples were reduced with DTT (15 mM final concentration, 30 min at 60°C) and alkylated with IAA (5 mM final concentration 30 min, room temperature in the dark). Digestion with trypsin was carried out over night and stopped by acidification (0.05% TFA final concentration). Peptide concentration was determined by amino acid analysis as described in^7^ and 200 ng of peptides were used for MS analysis.

The nanoHPLC analysis was performed on an UltiMate 3000 RSLC nanoLC system (Thermo Fisher Scientific, Bremen, Germany) as described in^8^. A 1 h washing step was used for internal equilibration of the column after each measured sample. The HPLC system was online-coupled to the nanoESI source of an Orbitrap Elite mass spectrometer (Thermo Fisher Scientific). In the ESI-MS/MS analysis, full MS spectra were scanned in a range between 350 and 2000 m/z with a resolution of 60,000 at 200 m/z for the detection of precursor ions. The spray voltage was set to 1,600 V (+), and the capillary temperature to 275°C. The top 20 most intensive ions (charge state +2, +3, +4) were selected for fragmentation experiments and their respective precursors were set on a dynamic exclusion list for 30 s.

MS/MS fragments were generated via collision-induced dissociation (CID) with a normalized collision energy (NCE) of 35 and an isolation window of 2.0 m/z. The produced fragments were analyzed in an iontrap with a resolution of 5.400 MS^2^.

The data analysis was carried out as described in^9,10^ using Progenesis Software (Progenesis QI for Proteomics, Nonlinear Dynamics Ltd., Newcastle upon Tyne, UK) for ion intensity based label-free quantification. Briefly, raw files were imported and aligned in Progenesis and simultaneously searched by Proteome Discoverer (ver 1.4.1.14) and the Mascot Search Engine (version 2.5) using the SwissProt part of the UniProtn^11^ KB for *homo sapiens*. DecoyDatabaseBuilder was used for the generation of shuffled decoy entries for each protein^12^. Mass tolerance was set to 10 ppm on precursor level and 0.4 Da on fragment ion level. As variable modifications, oxidation of methionine was set, and carbamidomethylation of cysteine was set as fixed modification due to sample preparation. One tryptic miscleavage was considered in the analysis. The peptide identifications were then matched to the respective features in Progenesis. FDR estimation was carried out for each search separately using Protein Inference Algorithm (PIA)^13-15^ by determining Mascot Ion Score thresholds at 1 % FDR. Proteins quantified with an ANOVA p-value < 0.05 were considered to be significantly differentially expressed in one of the sample types and were further used for the evaluation of specific cardiac markers, which verified a high quality of iPSC transduction into cardiomyocytes.

**LDH Cytotoxicity Assay**

An LDH assay was conducted with CyQUANT™ LDH Cytotoxicity Assay (Thermo Fisher #C20300) for SFS.1-CVB3ΔVP0-IRES-Venus#9 to determine the cytotoxic effect of caged-doxycycline (c-dox) and UV illumination. First, the appropriate cell concentration for the assay by which the assay is still in the linear range was determined by seeding SFS.1-CVB3ΔVP0-IRES-Venus#9 in 96-wells as triplicates in the concentrations 0, 250, 500, 1,000, 2,500, 5,000, 7,500, and 10,000 cells/96-well in FTDA-medium with 10 μM Y-27632. The next day medium was changed to FTDA without Y-27632. After two days of culture, the LDH assay was conducted according to manufacturer’s instructions. The appropriate cell concentration was determined to be at 1000 cells per 96-well. Next, cells were seeded two days prior to the LDH assay and shortly before the assay, cells for the UV assay were illuminated in a dry state to prevent light scattering for 1, 3, and 6 min and were added new media as well as the other cells. The maximum LDH release control was treated for 45 min with lysis buffer as described in the manufacturer’s instructions and within 30 min of incubation c-Dox (10 mg/ml in DMSO) was substituted to the respective cells at a concentration of 2 µg/ml (0.02% DMSO), 20 µg/ml (0.2% DMSO), and 200 µg/ml (2% DMSO). After 15 min the assay was conducted according to the manufacturer’s instructions.

**Life/dead staining**

The potential cytotoxic effect of doxycycline treatment on SFS.1-CVB3ΔVP0-IRES-Venus#9- and on SFS.1wt-derived cardiomyocytes was measured with life/dead staining. For sample preparation, differentiated cardiomyocytes on day 8 of differentiation were detached and reseeded in a ratio of 1:6 into matrigel coated 24 wells and cultivated for 5 more days. On day 14 of age, the treatment with 2 μg/ml doxycycline started. After 7, 14 and 21 days, cells were prepared for the life/dead staining. Seahorse XF96 plates (Agilent) were coated with 1:75 diluted matrigel and 0.2% gelatin solution prior to cell seeding. Differentiated cardiomyocytes were detached and pelleted as described before and seeded into the pre-coated plates in a density of 30,000 cells/well in 100 µL KO-THAI medium + 10 μM Y-27632. The next day, medium was changed to KO-THAI without Y-27632. After another 24h medium was removed and the cells were washed once with PBS. New KO-THAI medium containing NucGreen™ Dead 488 ReadyProbes™ Reagent (Thermo Fisher) and Hoechst33342 (Abcam) following the manufacturer´s instructions was added to the cells. After an incubation time of 5 min, the Seahorse XF96 plate was inserted into the Cytation1 multi-mode Imaging reader (Agilent). For descent imaging of single cells, preferred particle size was set to 5-50 μm. All seeded wells were imaged and analyzed by the Cytation1. Cell counts and ratio of life/dead cells was determined by the Gen5 Image Software. The acquired data were exported into an excel file and analyzed with OriginPro.

**Localized CVB3ΔVP0 expression via caged doxycycline**

SFS.1-CVB3ΔVP0-IRES-Venus cells were induced by caged-doxycycline in DMSO. For documentation of highly localized expression, cells were previously seeded on 12 mm glass cover slips coated with 1:75 diluted Matrigel^®^ and 0.2% gelatin in a ratio of 1:1. For localized induction, caged-doxycycline was added at 200 µg/ml to the respective culture medium under UV-protected conditions and incubated for 15 min at 37°C and 5% CO_2_. Subsequently, the cells were washed twice with PBS and their respective culture medium was added to the cells. Cells were UV-radiated 3 min each inside wells allowing for patterned UV irradiation to release doxycycline locally and thereby induce local CVB3 expression (Fig. 3b, 3c, and supplementary figure 1). After one day, the cells were fixed with 4% PFA/PBS and viewed under a confocal microscope at 515 nm excitation. A versatile custom UV-illumination capsule was designed (Fig. 3c). A standard laser lens and a round silica glass (1 mm thick) was inserted from the outside and glued to a 3D-printed corpus. A 1W-UV-LED was glued on a round heat sink using a thermally conductive paste and the heat sink was glued to the 3D-printed corpus from the other side. Wires soldered to the LED were guided through a hole in the corpus. The respective hole was closed with super glue to completely encapsulate the LED making it water proof. Thus, the LED UV-illumination unit can be operated in incubators as well. The LED was driven by LED driver at a 3.7 V battery. The stl-files for 3D-printing of wells (infection and striped pattern) and UV-illumination unit can be downloaded from the online methods.

1 Zhang, M. *et al.* Recessive cardiac phenotypes in induced pluripotent stem cell models of Jervell and Lange-Nielsen syndrome: Disease mechanisms and pharmacological rescue. *P Natl Acad Sci USA* **111**, E5383-E5392, doi:10.1073/pnas.1419553111 (2014).

2 Frank, S., Zhang, M., Scholer, H. R. & Greber, B. Small Molecule-Assisted, Line-Independent Maintenance of Human Pluripotent Stem Cells in Defined Conditions. *Plos One* **7**, doi:ARTN e4195810.1371/journal.pone.0041958 (2012).

3 Piccini, I. *et al.* Adrenergic Stress Protection of Human iPS Cell-Derived Cardiomyocytes by Fast K(v)7.1 Recycling. *Front Physiol* **8**, doi:Artn 70510.3389/Fphys.2017.00705 (2017).

4 Hanson, P. J. *et al.* Cleavage of DAP5 by coxsackievirus B3 2A protease facilitates viral replication and enhances apoptosis by altering translation of IRES-containing genes. *Cell Death Differ* **23**, 828-840, doi:10.1038/cdd.2015.145 (2016).

5 Lloyd, R. E. Enterovirus Control of Translation and RNA Granule Stress Responses. *Viruses-Basel* **8**, doi:Artn 9310.3390/V8040093 (2016).

6 Liberman, N. *et al.* DAP5 associates with eIF2 beta and eIF4AI to promote Internal Ribosome Entry Site driven translation. *Nucleic Acids Res* **43**, 3764-3775, doi:10.1093/nar/gkv205 (2015).

7 Guntermann, A. *et al.* Human tear fluid proteome dataset for usage as a spectral library and for protein modeling. *Data Brief* **23**, doi:ARTN 10374210.1016/j.dib.2019.103742 (2019).

8 Maerkens, A. *et al.* New insights into the protein aggregation pathology in myotilinopathy by combined proteomic and immunolocalization analyses. *Acta Neuropathol Commun* **4**, 8, doi:10.1186/s40478-016-0280-0 (2016).

9 Oertzen-Hagemann, V. *et al.* Effects of 12 Weeks of Hypertrophy Resistance Exercise Training Combined with Collagen Peptide Supplementation on the Skeletal Muscle Proteome in Recreationally Active Men. *Nutrients* **11**, doi:10.3390/nu11051072 (2019).

10 Winter, L. *et al.* Mutant desmin substantially perturbs mitochondrial morphology, function and maintenance in skeletal muscle tissue. *Acta Neuropathol* **132**, 453-473, doi:10.1007/s00401-016-1592-7 (2016).

11 Apweiler, R. *et al.* UniProt: the Universal Protein knowledgebase. *Nucleic Acids Res* **32**, D115-119, doi:10.1093/nar/gkh131 (2004).

12 Reidegeld, K. A. *et al.* An easy-to-use Decoy Database Builder software tool, implementing different decoy strategies for false discovery rate calculation in automated MS/MS protein identifications. *Proteomics* **8**, 1129-1137, doi:10.1002/pmic.200701073 (2008).

13 Turewicz, M. *et al.* BioInfra.Prot: A comprehensive proteomics workflow including data standardization, protein inference, expression analysis and data publication. *J Biotechnol* **261**, 116-125, doi:10.1016/j.jbiotec.2017.06.005 (2017).

14 Uszkoreit, J. *et al.* PIA: An Intuitive Protein Inference Engine with a Web-Based User Interface. *J Proteome Res* **14**, 2988-2997, doi:10.1021/acs.jproteome.5b00121 (2015).

15 Uszkoreit, J., Perez-Riverol, Y., Eggers, B., Marcus, K. & Eisenacher, M. Protein Inference Using PIA Workflows and PSI Standard File Formats. *J Proteome Res* **18**, 741-747, doi:10.1021/acs.jproteome.8b00723 (2019).
